# A multi-purpose pilot-scale molten metal & molten salt pyrolysis reactor

**DOI:** 10.1016/j.mex.2021.101606

**Published:** 2021-12-16

**Authors:** Frank Riedewald, Ian Povey, Maria O'Mahoney, Maria Sousa-Gallagher

**Affiliations:** a Composite Recycling Ltd, The Rubicon Centre, CIT Campus, Bishopstown, Cork T12 Y275, Ireland; b Tyndall National Institute, University College Cork, Lee Maltings, Dyke Parade, Ireland; c Environmental Research Institute, University College Cork, Ireland; d Process and Chemical Engineering, School of Engineering, University College Cork, Ireland

**Keywords:** Pilot plant design, Operation, Recycling, Biomass, Composite plastic waste

## Abstract

This paper describes the design features and operational details of a molten metal pyrolysis reactor. Such a reactor allows pyrolysis experimentation on biomass, aluminium-laminated plastics, mixed plastics, carbon fibre materials, etc. Experimental results on biodegradable plastic, carbon fibre composites, biomass and printed circuit boards (PCBs) are presented.

• The inner container can have a sloped or flat-bottom depending on the material.
• The method can be used to pyrolyse composite and pure materials.

## Graphical abstract

**Figure:**
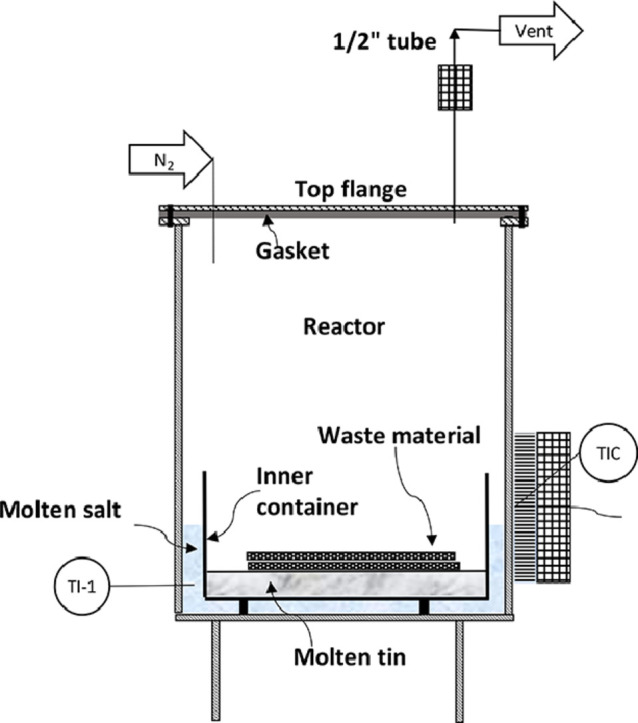

Specifications Table

**Table:** 

Subject Area:	Chemical Engineering
More specific subject area:	Pyrolysis reactor system
Method name:	Recycling of waste plastic and waste composite plastic
Name and reference of original method:	Not applicable
Resource availability:	Not applicable

## Background

Pyrolysis is a process capable of recycling many materials such as mixed plastics [1], [2], [3], aluminium-laminated plastics or Tetra Pak [4], glass or carbon fibre plastic composites [5], automobile shredder residue [6], whole tyres [7] and lithium-ion batteries [8]. Moreover, pyrolysis is used to produce bio-oil from biomass [9]. Pyrolysis is a depolymerisation process executed at temperatures above 400°C, in an oxygen-free environment and, typically, at ambient pressures [10]. Products of mixed plastic pyrolysis are pyrolysis oil (ca. 80-95%), pyrolytic carbon (ca. 1-5%; also referred to as char or ash in the literature) and gases (5-15%) depending on the pyrolysis temperature, catalyst, and the mixed plastic composition [11,12].

The emphasis of this research is on the solid residue left inside the reactor. For several important waste streams, for instance, lithium-ion batteries or carbon fibre material, the solid residue is the main revenue stream for a pyrolysis process operator.

The composition and yields of the pyrolysis oil and gasses are known from previous work. The gas and oil pyrolysis composition for various plastics is given in [11,12]. In contrast, [13,14] gives the same for carbon fibre pyrolysis, whereas [15,16] presents the oil and gas data for lithium-ion batteries and [17], [18], [19], [20] gives the same for biomass. Therefore, this research concentrated on the solid residues instead of collecting and analysing the pyrolysis oil and gases or establishing the yields.

### System description

**Fig. 1:**
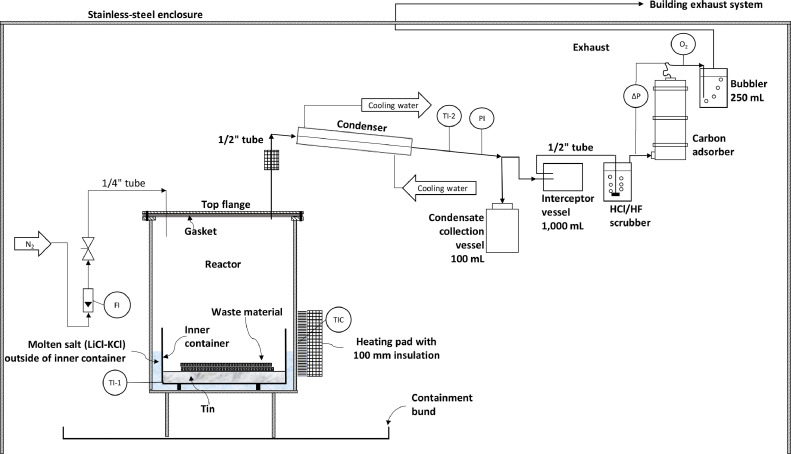
Process and instrumentation diagram of the multi-purpose pyrolysis; (TI-1 = temperature indicator reactor, TI-2 = temperature indicator condenser outlet pipe, TIC = temperature indicator controller, PI = pressure indicator, ∆P = differential pressure indicator, FI = N_2_ flow indicator, O_2_ = handheld oxygen monitor, N_2_ = nitrogen).

**Fig. 2:**
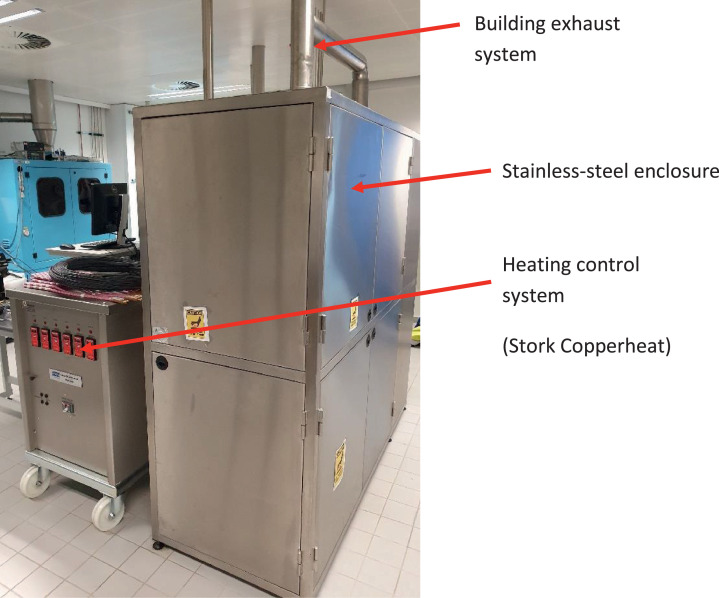
View of the stainless-steel enclosure housing the experiment.

**Fig. 3:**
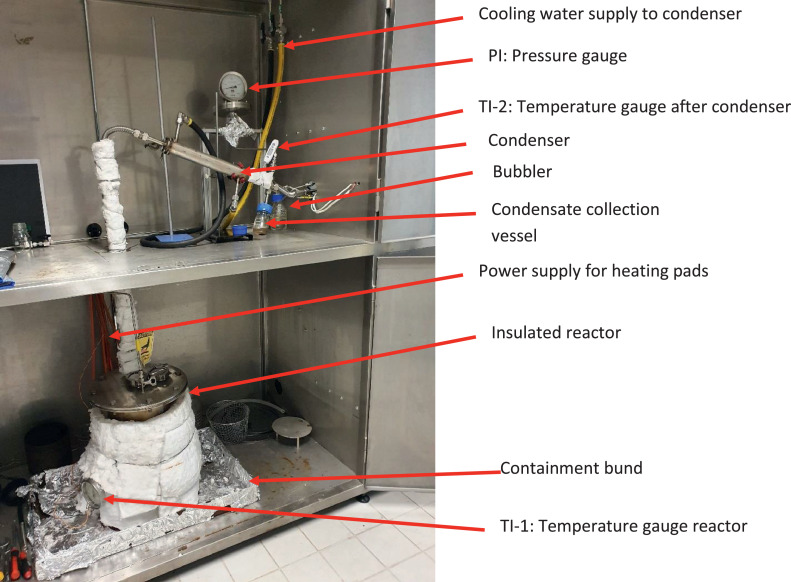
View of the experiment with the stainless-steel enclosure doors open as during the execution of an experiment.

This paper presents a pilot-scale multi-purpose pyrolysis system. The process and instrumentation diagram is given in Fig. 1 and shows the various parts of the experimental system described below. The experiment is placed within a stainless-steel enclosure (Fig. 2). The reactor (Fig. 3) is manufactured from an 8” (125 mm; i.e., the diameter of the reactor) ANSI schedule 10, 316L stainless steel pipe. The reactor has a height of 300 mm. A 3 mm thick 316L stainless steel plate is the bottom wall of the vessel. The vessel can be opened by unbolting the top flange, which was cut from a 3 mm thick 316L steel plate.

The reactor is heated by three electrical heating pads (ceramic pad heating elements up to 800°C supplied by Stork Copperheat, UK). Two pads are wrapped around the lower part of the reactor, and the third one is located below the bottom plate. The vessel is insulated by 100 mm glass wool (supplied by Stork Copperheat, UK). But the top flange and the vessel sides below 80 mm of the flange are not insulated. An Ashcroft temperature gauge (range of 0 to 500°C, with a 1% ASME B40.3, Grade A accuracy inserted into a thermowell manufactured from 316L stainless steel) is used to measure, but not to control, the operating temperature. The operating temperature is controlled to an estimated ±5°C by a control system (Stork Copperheat, UK, 50 KVA Heat Treatment Module, Model no. 16050 shown in Fig. 2). A thermocouple located between the outside surface of the reactor and one of the heating pads (TIC, Fig. 1) provides the temperature feedback to the control system.

The inner container can be changed, allowing experimentation on various feedstock such as printed circuit boards, tyres, automobile-shredder residue, aluminium-laminated plastics, lithium-ion batteries or biomass. Moreover, depending on the feedstock, pyrolysis experiments with molten metal or molten salt can be executed. The inner container can also have different shapes and configurations; for instance, it could have a sloped bottom. Fig. 1 shows a 6” 316 L stainless-steel flat-bottom inner container, which was filled with 1,300 g of tin, equating to a molten tin level of 1 cm. This flat bottom inner container was used for the experiments shown in Fig. 6, Fig. 7, Fig. 8.

The space between the reactor walls and the inner container is filled with 2,600 g of salt, providing heat transfer between the reactor walls and the inner container. This salt is a eutectic mixture of technical grade lithium chloride (LiCl) and potassium chloride (KCl) salts (41.8 mol% KCl and 58.2 mol% LiCl) having the relevant physical properties given in Table 1 in its molten state at 450°C. LiCl-KCl salt was chosen for these experiments, as it is stable, non-toxic and inert at the operating temperatures [21]. Additionally, 316L stainless steel is a suitable material of construction for molten LiCl-KCl [22].

**Table 1:** Relevant physical properties of molten tin and molten LiCl-KCl at 450°C compiled from [21,25].

Compound	Density [kg/m^3^]	Melting point [°C]	Vapour pressure [Pa]
LiCl-KCl (molten)	∼1,600	355	133 at 800°C
Tin (molten)	6,990	232	1.26-10^−9^ at 630°C

As the inner container is heated via a heat transfer salt, a large temperature gradient within the reactor is likely. Hence, TI-1 (Fig. 1 & 3) may not indicate the actual temperature within the inner container. Therefore, during the commissioning phase, heat-sensitive paints (Tempilaq Temperature Indicators paints with temperature changing points of 900°F, 850°F, 800°F and 750°F, supplied by Walters & Walters, UK) applied on a 40 mm long, 10 mm wide and 10 mm high 316L stainless steel boat was used to calibrate the temperature gauge TI-1 to the temperature within the inner container. The boat was secured by a wire so that it was held in the middle of the inner container floating, simulating a feedstock. Measuring the molten metal or salt temperature in the inner container with a temperature gauge supported from the top flange is not straightforward. During cooldown, the molten material would solidify around the probe, thus locking the temperature gauge in place and, as a result, preventing the opening of the reactor.

The pressure gauge (PI, Fig. 1 & 3) is a high accuracy, low-pressure instrument (Stewart-Buchanan Gauges Ltd, UK, model 2012, 4” dial mechanical diaphragm gauge with a 316 stainless steel diaphragm unit, accuracy ± 2% of full scale, range 0-400 mbar).

The gasket sealing the top flange of the reactor is a 3 mm thick, full face blank graphite laminate gasket, custom cut to suit the reactor, with a maximum operating temperature of 500°C. However, the maximum temperature the top flange reached is only 130°C, measured with a GenWare infrared-thermometer (range: -32 to 550°C with a 0.5 second response time).

The preferred pipe connections are metal-to-metal compression fittings (Swagelok) as they are vapour tight and can be opened for inspection. All high temperature (over 100°C) pipes are ½” Swagelok 316L tubes with metal-to-metal compression fittings. The pyrolysis vapours are condensed by a 30 cm long section of the ½” tube, which is cooled with ambient water. The condenser slopes by 30 degrees and drains into a 100 mL condensate collection vessel (Fig. 3).

A temperature gauge (TI-2, Fig. 1) at the condenser outlet measures the temperature of the vapour stream and, hence, indicates the efficiency of the condenser. A temperature increase of 2-3°C was observed at very high organic vapour loading, i.e., at the beginning of the pyrolysis process, dropping back to ambient shortly afterwards. But if the temperature rose by 5-8°C, water was present, which showed up as a separate phase in the condensate collection vessel. A 5-8°C temperature rise was always associated with fresh heat transfer salt (LiCl-KCl), which is always slightly wet even if appearing dry. It also demonstrates that it is essential to keep the salt free of water, i.e., seal the reactor and replace the air with nitrogen during idle times.

The pipe connecting the reactor to the condenser is insulated to minimise condensation in the line leading to the condenser, which would result in reflux to the reactor.

**Fig. 4:**
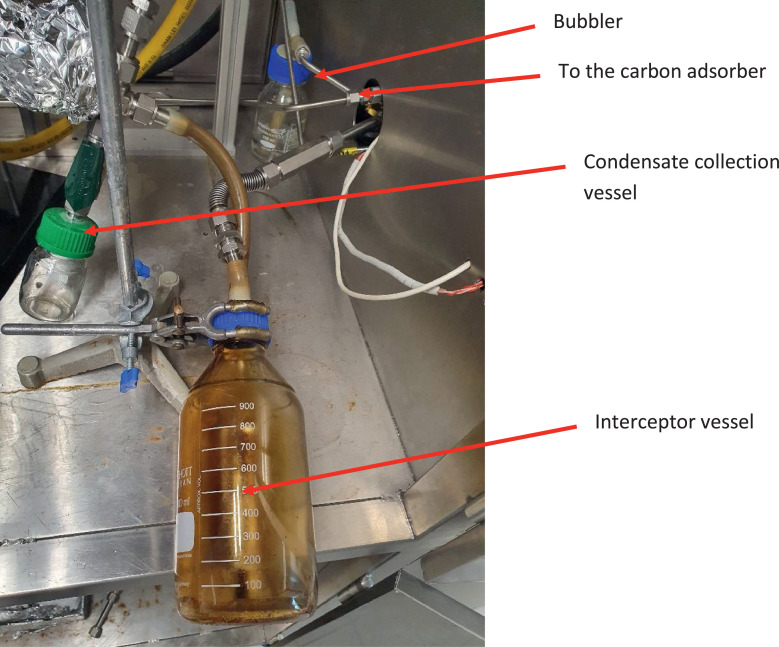
Piping setup of the interceptor vessel. Schott GL45 top with two connections (screw top made suitable for hose to slip on with resin glue, ½” tube with resin from both sides.

Polyolefin pyrolysis may result in copious amounts of waxes. For instance, at a pyrolysis temperature of 450°C, polypropylene generates up to 92% waxes [4,^11^,^12^,23], which have the potential to block the condenser [24] or other pieces of equipment if these are not designed for waxes. Moreover, sharp 90-degree bends are best avoided as waxes suspended in the vapour stream collect there. Sweeping bends, as shown by the line connecting the condenser to the interceptor vessel (Fig. 4), were successful in avoiding waxes from accumulating in a pipe bend and blocking it.

**Fig. 5:**
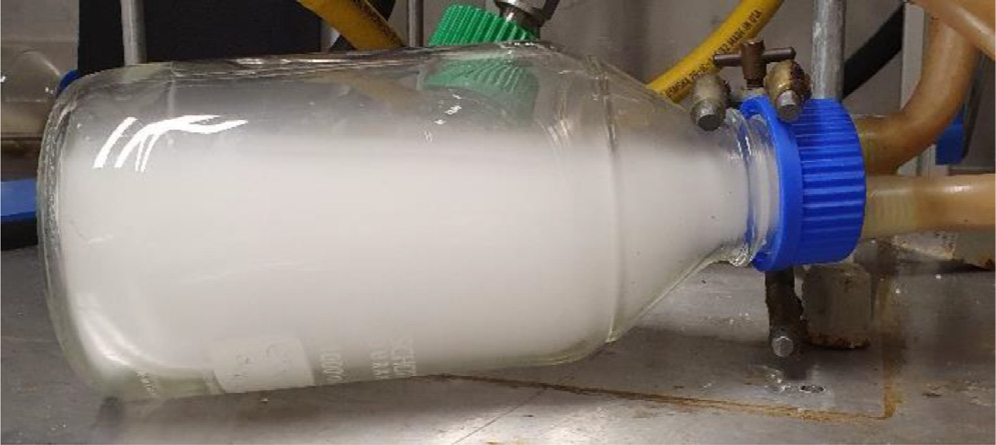
Photo of the interceptor vessel during one of the experiments on aluminium-laminated plastics.

A 1,000 mL laboratory glass bottle, named interceptor vessel (Fig. 1 & 4), gives a visual impression of the vapour stream after the condenser. The fluid velocity reduces within this vessel, allowing waxes and other particles suspended in the vapour stream to sink, as visible in Fig. 5, showing the top section of the vessel clear of fog. Silicone hoses (APCST/16 × 22.5 silicone tubing 16 mm bore x 22.5 mm O.D., 3.25 mm wall thickness suitable for a temperature of up to 200°C) are used to connect the interceptor vessel to the ½” tubes. A GL45 top is used to connect the interceptor vessel to the silicone hoses and the ½” tubes (screw top made suitable for the silicon hose to slip on and be leak-tight with resin glue). The operating temperature is ambient at this point; hence silicone hoses instead of 316LSS pipes can be used.

A carbon adsorber filled with 25 kg activated carbon (Silcarbon Aktivkohle GmbH, Germany) abates the vapour stream from the experiment before discharging it to the air extract system of the building. A pressure differential measurement (∆P, Fig. 1) across the carbon adsorber indicates if the carbon adsorber is nearing its capacity (Stewart-Buchanan Gauges Ltd, UK, model 4901/PCS, 4” dial differential pressure gauge, 316 stainless steel case and wetted parts, accuracy ± 2% of full scale, range 0-100 mbar).

### System operation

The system is operated in the following sequential steps: 1. Load the reactor and close the vessel; 2. Inert the experiment with nitrogen and leak test the system; 3. Heat the reactor to the operating temperature of 450°C; 4. Maintain the operating temperature for 60 minutes; 5. Shutdown and cool down; 6. Remove the pyrolysed material from the reactor for analysis.

Before any pyrolysis experiment is carried out, the system, including the carbon adsorber, is nitrogen-sweep inerted for a minimum of 30 minutes at a flow rate of 300 norm litres per hour (Nl/h). The inert state of the system is confirmed by a handheld oxygen analyser (B.W. Technologies, GasAlert Extreme s/n J615-X043713), which must indicate that the oxygen level is below 0.1% before proceeding to the pyrolysis phase. It is measured after the carbon adsorber by disconnecting a hose before the bubbler (O_2_, Fig. 1). During any experiment, the formation of an explosive atmosphere within the pyrolysis vessel vapour space and downstream equipment is prevented by continuously sweeping the system with a nitrogen flow of 50-100 Nl/h.

The operating pressure of the entire system is slightly above atmospheric (∼10 mbar), preventing air ingress due to the hydrostatic pressure induced by a bubbler. The bubbler is a 250 mL laboratory glass bottle filled with 100 mL of water through which the nitrogen flow is directed, making the nitrogen flow through the experiment visible (Fig. 1,3 & 4).

The top flange gasket and all other connections which are opened (e.g., the bottles) are tested with soapy water for leaks during the inertion phase. Should a leak be detected, the experiment should not proceed. Some problems encountered to seal screw connections at the carbon adsorber were made leak-tight by applying Epoxy adhesive onto them (Gorilla epoxy) sealing them completely.

For the pyrolysis experiments, an operating temperature of 450°C was chosen. Once this operating temperature was reached, it was maintained for 60 minutes to ensure the pyrolysis reaction was completed. On reaching this point, the heat was turned off, and the equipment cooled naturally to ambient. By removing the insulation, the cooldown period may be reduced. When the reactor cooled down to 100°C, the nitrogen flow was switched off. Finally, the reactor was opened if the reactor temperature was less than 40°C, and the pyrolysed material was removed for analysis.

Depending on the loading of the reactor, i.e. the type of the inner container, the electrical heating system achieved an average temperature rise of 9-11°C per minute (calculated between ambient and the operating temperature of 450°C).

The molten metal is tin. Tin is non-toxic, melts at 232°C and boils at 2,602°C [25], offering an extensive operating range. Moreover, 316L stainless steel is a suitable material of construction for molten tin for the operating temperature of these experiments [26]. Corrosion of the stainless steel by molten tin is, however, restricted to the inner container. Visual inspection of the inner container after twelve experiments with molten tin did not reveal any corrosion.

LiCl-KCl salt is a deliquescent salt, i.e. it absorbs so much moisture from the atmosphere that it dissolves in it, forming a solution. This very property of the salt can be used to remove the inner container, as the salt must be dissolved before the inner container can be removed from the reactor.

A future addition to the experiment may be a caustic scrubber to remove acids such as HCl or HF from the vapour stream because the pyrolysis of, for instance, polyvinyl chloride generates HCl [11], which a caustic scrubber would remove.

After every experiment, the condenser and the pipe leading and discharging from it were inspected for evidence of waxes or other deposits. And after ten experiments, the reactor was visually inspected for any evidence of corrosion; none was found.

During an experiment, the enclosure doors are open, as the bubbler and the pressure and temperature readings must be observed at all times.

The operator wears personal protective equipment (PPE) suitable for the foundry industry during any pyrolysis experiments. The PPE is aluminised protective clothing and conforms to EN ISO 11612.

### Method validation

This research aims to improve the recycling of printed circuit boards, lithium-ion batteries, automobile shredder residue, and aluminium-laminated plastics. But before any experiments on these materials were executed, the pilot plant operation was checked for leak-tightness and general performance on biomass and carbon fibre materials.

**Fig. 6:**
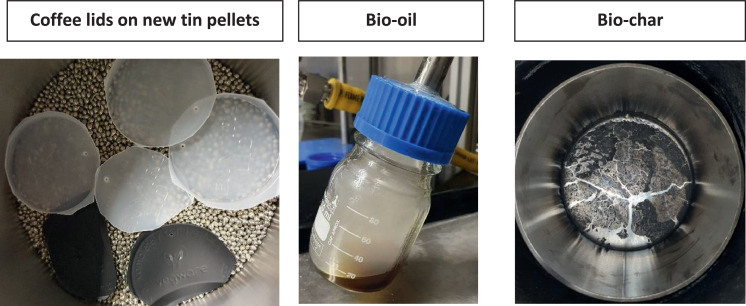
Results of a biodegradable plastic coffee lids pyrolysis experiment on molten tin.

Biomass is relatively easy to pyrolyse, as the vapour stream does not contain any waxes. Moreover, no toxins are generated, nor is the smell of the pyrolysis gas too pungent should a leak occur during testing as sulphur or brominated compounds are not present [20]. Therefore, biomass, i.e., biodegradable plastic coffee lids (Fig. 6), was used to test the system for the first set of experiments. These experiments proved that the system was leak-tight. Moreover, it proved that the condenser worked as the amount of bio-oil collected was broadly in line with the data given by [20] on biomass pyrolysis.

**Fig. 7:**
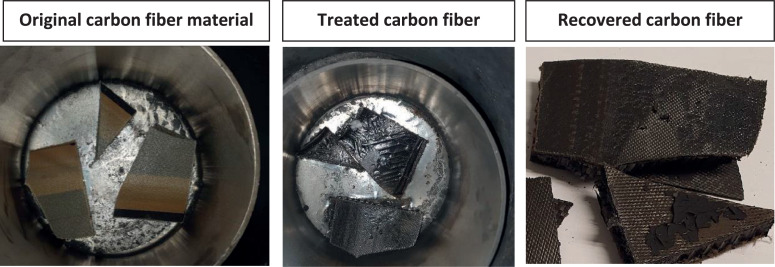
Carbon fibre material (aircraft part) pyrolysed on molten tin.

The second set of experiments on carbon fibre resin (Fig. 7) introduced a more challenging material from a leak point of view, i.e., a material that causes a pungent smell should a leak occur or should the carbon adsorber not working correctly. Giorgini et al. [14] report that hardly any pyrolysis oil is produced by carbon fibre pyrolysis – a result confirmed by the carbon fibre pyrolysis experiments on molten metal. Moreover, the 100 mL condensate collection vessel did not collect any water. Thus, these experiments demonstrated that the heat transfer salt remained dry, although the system was idle over a weekend.

Biomass, that is, beechwood, was also used to test the system after changes were introduced. The interceptor vessel, for example, was installed to inspect the vapour stream after the condenser visually. It was, however, not part of the original setup. This interceptor vessel demonstrates that the condenser did not entirely remove waxes generated from the pyrolysis of polyolefins. Instead, tiny wax droplets stayed suspended in the vapour stream, as shown in Fig. 5. In addition, the interceptor vessel was not always cleaned between experiments avoiding opening the system. Hence the brown deposits on the glass surface as visible in Fig. 4.

**Fig. 8:**
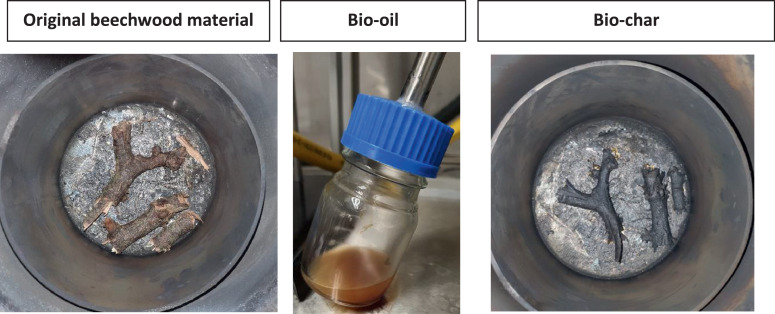
Results of a beechwood pyrolysis experiment on molten tin.

The experiments on biodegradable plastics, carbon fibre and beechwood biomass demonstrated that molten tin repels carbon or silicon oxide, meaning that these materials float on the molten metal and can be recovered [27], although they were in direct contact with the molten tin. The biochar (Fig. 8, right), for example, can be removed whole from the solidified tin without removing any tin or leaving char behind.

**Fig. 9:**
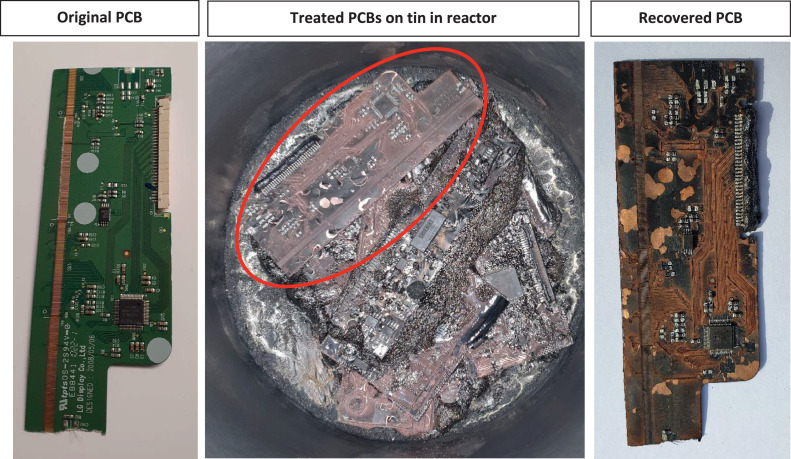
Results of pyrolysis of PCBs on molten tin. Left: original PCB, middle: the situation after opening the cooled down reactor (circle shows the PCB on the right), right: recovered PCB.

Fig. 9 shows images of a PCB before, in the reactor after treatment and recovered from the reactor. No evidence from any PCBs treated is available showing that any PCB metals dissolved in the molten tin, although molten tin alloys with copper, gold and other metals [28]. The reason for this failure seems to be that the metallic PCB compounds did not come into contact with the molten tin as a layer of carbon or glass fibre prevented contact. Moreover, it was no problem to remove the treated PCBs from the reactor.

## Conclusion

This work presents a batch operated, multi-purpose pyrolysis system using molten metal or molten salt. This new reactor setup improves the operational capabilities of the earlier molten salt [29] and molten metal [30] experimental systems. Moreover, using various inner containers, different reactor conditions (sloped bottom, salt and metal or metal or salt only) may be investigated. From a safety perspective, two improvements were made to the earlier version of the experiment [29,30]. First, open flames are avoided and second, the operator is no longer exposed to molten metal or molten salt surfaces as the molten materials are contained within the reactor. Hence, this system allows the safe and fast pyrolysis of various feedstocks, including composite plastics. Furthermore, with this system, feedstock recycling with molten metal or molten salt, or both can be assessed.

It is possible to upgrade the reactor system to obtain yield data for the various wastes. For instance, the top flange of the reactor reached only 130°C, waxes condensed there, as was found on inspection of the reactor after an aluminium-laminated plastic experiment. Insulating the flange and maybe heat tracing along with the pipe to the condenser would avoid waxes from condensing on those surfaces. But it would be essential to design the condenser system for waxes should polyolefin plastics be pyrolysed. Park et al. [24] provide details of how to design the condenser system for waxes.

Furthermore, it is good practice not to use a full load immediately. Instead, the amount of feedstock should be increased over time. Lastly, the system should be inspected for waxes or other deposits between any experiment.

## Declaration of Competing Interest

The authors declare that they have no known competing financial interests or personal relationships that could have appeared to influence the work reported in this paper.
